# Turning a phage anti-defense weapon into an immunity trigger

**DOI:** 10.1371/journal.pbio.3003978

**Published:** 2026-09-16

**Authors:** Anna B. Adelstein, Naama Aviram

**Affiliations:** Molecular Biology Program, Sloan Kettering Institute, Memorial Sloan Kettering Cancer Center, New York, New York, United States of America

## Abstract

Most bacterial immune systems raise an alarm when they sense a phage. A new study in PLOS Biology shows how type II Panoptes reverses this logic by maintaining a quiet signal and sensing infection through its sudden disappearance

In the last decade, our understanding of bacterial defense mechanisms against phages has surged with hundreds of independent defense systems discovered [1–4]. Within this “golden era” of bacterial immune research, many systems have been studied in isolation, with little insight into how they might work together to coordinate a robust immune response. A recent *PLOS Biology* study by Grüschow and colleagues [5] has characterized the molecular mechanism of type II Panoptes, a potential guard system for type III CRISPR anti-phage defense. Like the previously characterized type I Panoptes system, type II Panoptes constitutively generates a signaling nucleotide to keep a toxic effector switched off until it encounters phage infection, the reverse of typical nucleotide-mediated systems which switch on anti-phage signaling in response to infection (Fig 1A).

**Fig 1:**
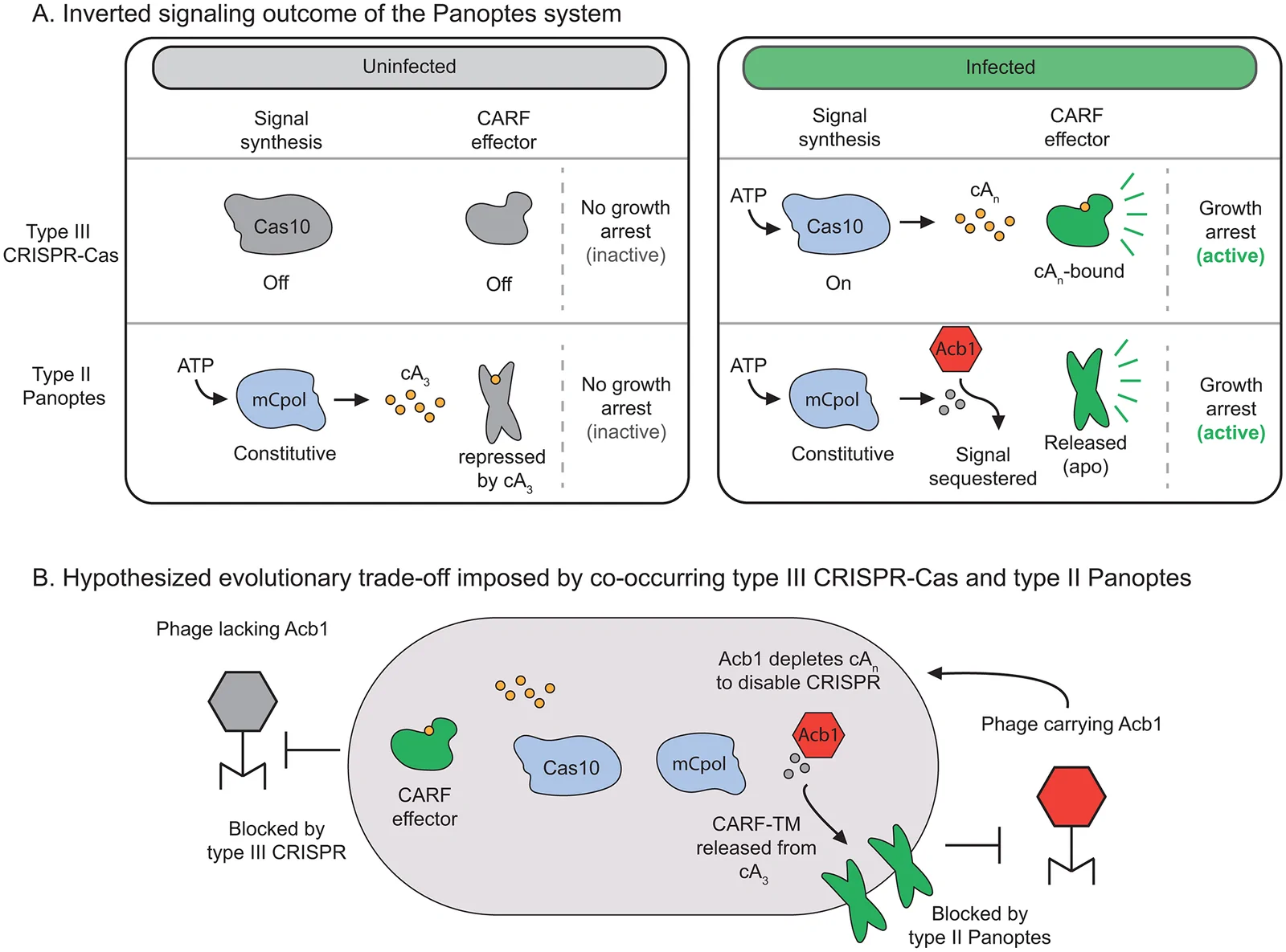
Type II Panoptes inverts the logic of nucleotide signaling in bacterial immunity. (A) In type III CRISPR-Cas, Cas10 synthesizes cyclic oligoadenylates (cA_n_) only upon infection; the signal binds and activates a CARF effector, arresting growth. Type II Panoptes does the reverse. Its mCpol enzyme produces cA_3_ constitutively at low levels, and this signal keeps the CARF-TM effector repressed. Infection is sensed not as the appearance of a signal, but as its disappearance: when phage enzymes (Acb1 given as an example) deplete cA_3_, the effector is released and growth arrests. **(B)** Because Acb1 evolved to disable cyclic-nucleotide defenses such as type III CRISPR, a phage carrying it strips cA_3_ as collateral damage and triggers Panoptes. A phage lacking Acb1 avoids this trap but remains vulnerable to CRISPR. Together, the two systems could impose an evolutionary trade-off on the phage.

In order to combat phages, bacterial defense systems must rapidly sense infection and signal to effector proteins to clear it. The study of multiple defense systems has revealed a trend in the use of cyclic oligonucleotides as important second messengers for the activation of anti-phage defense [6]. This strategy presents notably in both the CBASS system and the type III CRISPR system, among others. CBASS systems use a CD-NTase (cGAS/DncV-like nucleotidyltransferase) to generate cyclic nucleotide signals that bind the Cap effector which, once activated, induces cell death [7]. In type III CRISPR systems, the primary effector Cas10 cyclizes ATP into cyclic oligoadenylate molecules, cA_n_ (where *n* = 2–6), which signal to accessory proteins including CRISPR-associated Rossmann Fold (CARF) proteins [8]. These CARF proteins activate when they bind cA molecules and then induce cell growth arrest to suppress phage propagation [9]. To counter these defenses, bacteriophages have evolved to express proteins antagonistic to these second messengers, such as sponge proteins that sequester them, phosphodiesterases, and ring nucleases [5,10,11].

Panoptes systems constitutively generate cyclic oligonucleotides to *repress* their effector function through allosteric binding that affects the effector conformation [5]. These systems have been proposed to act as guards and are frequently encoded alongside the systems they could protect. Roughly half of Panoptes systems share a genome with a CBASS system, and half of those sit within the same gene neighborhood [10,11]. Panoptes is also significantly enriched in genomes carrying type III CRISPR [5]. Sensitive to cyclic oligoadenylate depletion, Panoptes activates when phage proteins degrade or sequester its decoy oligonucleotide, releasing the repressed and toxic effector, which staunches phage propagation by inducing cell death or growth arrest [5,10,11].

Panoptes guard systems are relatively newly described systems in the field of bacterial defenses, with the type I Panoptes system being characterized just last year [10,11]. The type I system encodes a Cas10-like protein called mCpol (minimal CRISPR polymerase), which constitutively generates 2′3′-c-di-AMP molecules from ATP. These signaling molecules bind the 2TMβ protein, a CBASS Cap15 homologue, disrupting its oligomerization and repressing its membrane-depolarizing function. Type I Panoptes provided the first example of a system that uses signaling nucleotides as negative regulators. Yet of the known Panoptes systems, 40% are of the type II clade [11]. Grüschow and colleagues are the first to characterize the type II Panoptes system, which represses the function of a CARF-like protein, revealing a potential guard mechanism for type III CRISPR.

The authors use the *Cylindrospermum stagnale* (Cst) model to first characterize the type II Panoptes system. They show that the Cst mCpol constitutively produces 3′-3′-3′ cA_3_ in the presence of ATP and Mg^2+^, exhibiting a similar function to the type I mCpol but generating a different signaling molecule, a result they reproduced in two other type II Panoptes systems. Notably, these mCpol enzymes turn over slowly, producing cA_3_ at low concentrations. Given the “reverse-logic” of Panoptes signaling, this fits the decoy mechanism: a guard does not need a strong signal, only a steady trickle sufficient to keep its effector off and to detect sudden drops as the phage sequesters the signal. That effector is CARF-TM, a transmembrane protein whose activation is repressed when bound to cA_3._ The authors show that cA_3_ binds CARF-TM with high affinity and specificity. When the signal is removed and the effector shifts to its apo, unbound form, it induces just enough toxicity to send the cell into growth arrest, consistent with the behavior of other CARF proteins [5].

The real test came when the authors challenged their model system with phages. Among BASEL phages carrying the anti-CBASS protein Acb1, only those with a functional enzyme activated Panoptes. This is the heart of the guard strategy. Acb1 is the phage’s own weapon, which can dismantle CBASS and CRISPR by depleting their signaling nucleotides. Against a cell carrying Panoptes, that same weapon becomes a liability: in clearing away cyclic nucleotides, Acb1 also strips the cA_3_ decoy, releasing the effector the phage was never meant to wake [5]. In a bacterium carrying both Panoptes and the defenses targeted by Acb1, this could leave the phage with no good option: retaining Acb1 could activate Panoptes, whereas losing Acb1 could restore susceptibility to the defenses it counters.

Phages are thought to carry Acb1 and other signal-depleting systems primarily to counter defenses such as CBASS and type III CRISPR, not Panoptes. Panoptes may therefore exploit a weapon the phage already brought (Fig 1B). Consistent with this proposed model, Panoptes systems are often found alongside CBASS and type III CRISPR, though they can also defend effectively in bacteria carrying no other cyclic-nucleotide system. This coexistence raises an exciting question: whether and how these bacterial defense systems work together during phage infection. Under this model, co-occurrence would impose a subtle constraint: the decoy would need to be different enough from the cell’s genuine defense signals, that it does not set off the wrong alarm, yet close enough to what phages target that a phage attacking its neighbors cannot help but strip it away. Such functional interactions are not unique to Panoptes: previous studies have described synergy between co-occurring defense systems [12], restriction-modification-assisted CRISPR adaptation [13], and activation of PARIS by a phage anti-restriction protein [14].

Whether Panoptes and its co-localized CBASS and type III CRISPR systems act independently, or in a coordinated sequence, remains an open question, and one worth pursuing. Type II Panoptes therefore provides a compelling mechanistic model for how one defense system could exploit phage counter-defense directed at another.

## Abbreviations:

CARF: CRISPR-associated Rossmann Fold
CBASS: Cyclic oligonucleotide-Based Anti-phage Signaling System
CRISPR: Clustered Regularly Interspaced Short Palindromic Repeats
